# Chemical Profiling and In Vitro Assessment of Antioxidant and Antimicrobial Properties of Algerian *Erica arborea* L. Extracts, Strengthened by Molecular Modeling Analysis

**DOI:** 10.1002/open.70271

**Published:** 2026-08-04

**Authors:** Amina Bramki, Ouided Benslama, Fatima Zohra Makhlouf, Anna Andolfi, Zineb Mokhbi, Selsabile Nedjaoum, Chawki Bensouici, Maria Michela Salvatore, Marco Masi

**Affiliations:** ^1^ Higher National School of Biotechnology Taoufik Khaznadar Constantine Algeria; ^2^ Laboratory of Biological Engineering, Valorization, and Innovation of Agri‐Food Products University of Larbi Ben M’hidi Oum El Bouaghi Algeria; ^3^ Research Unit of Environmental and Structural Molecular Chemistry University of Constantine (Mentouri) Constantine Algeria; ^4^ Department of Chemical Sciences University of Naples Federico II Naples Italy; ^5^ Biotechnology Research Center Constantine Algeria; ^6^ Department of Veterinary Medicine and Animal Production University of Naples Federico II Naples Italy

**Keywords:** biological activities, *Erica arborea* L., molecular docking, phytochemical composition

## Abstract

This study aimed to valorize *Erica arborea* L. collected in Algeria by characterizing its chemical composition and assessing the biological potential of its organic extracts. The aerial nonflowering parts were extracted using solvents of increasing polarity, allowing evaluation of chemical and biological properties of extracts. Phytochemical assays revealed that the ethyl acetate (EtOAc) fraction contained the highest levels of total polyphenols (419.58 ± 1.89 µg GAE/mg), flavonoids (174.57 ± 1.93 µg QE/mg), and flavonols (61.64 ± 2.49 µg QE/mg). GC–MS profiling confirmed the presence of several bioactive constituents, supporting its rich chemical composition. Antioxidant activity highlighted the superiority of EtOAc extract, with IC_50_ of 5.07 ± 0.63 μg/mL in the ABTS^•+^ assay, 26.96 ± 1.16 μg/mL in the DPPH^•^ assay, and an A_0.5_ of 18.14 ± 0.57 μg/mL in the FRAP test, while the phenanthroline assay showed strong reducing power across all extracts (*A*_0.5_ = 1.25 ± 0.05–6.72 ± 0.15 μg/mL). Antimicrobial activity revealed that the EtOAc fraction exhibited notable activity against *Staphylococcus aureus* (19.33 ± 0.57 mm) and *Bacillus subtilis* (19.0 ± 1.0 mm), while all fractions showed antifungal activity against *Candida albicans* and *Fusarium oxysporum*. Molecular docking on xanthine oxidase and DNA gyrase (GyrA and GyrB) revealed favorable interactions of several metabolites, supporting the antioxidant and antibacterial activities observed experimentally.

## Graphical abstract

*Erica arborea* L. from Algeria was valorized via chemical profiling and bioactivity assessment. The EtOAc extract showed the highest levels of polyphenols, flavonoids, and flavonols. It exhibited strong antioxidant (ABTS•^+^, DPPH•, FRAP) and antibacterial activity (*S. aureus*, *B. subtilis*). Docking on xanthine oxidase and DNA gyrase supported these activities at the molecular level.

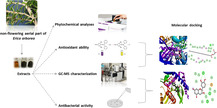

## 1 Introduction

Medicinal plants occupy a central place in traditional healthcare systems worldwide. Their use is rooted in a long‐standing empirical tradition that has contributed significantly to the discovery of numerous therapeutic agents and continues to play a fundamental role in modern pharmaceutical research [1]. These plant resources constitute a remarkable reservoir of secondary metabolites with diverse biological activities that have been exploited for centuries in traditional medicine. Over recent decades, scientific interest in medicinal plants has intensified, particularly due to the limitations of conventional therapies, including the increasing emergence of microbial resistance, the adverse effects associated with synthetic drugs [2], and the urgent need to identify new antioxidant agents capable of mitigating oxidative stress associated with many chronic diseases. In this context, the rational exploration of medicinal plants not only validates their traditional uses but also facilitates the identification of new molecules of pharmacological interest [3]. Among the botanical families recognized for their therapeutic potential, the Ericaceae family occupies a prominent position. This cosmopolitan family, comprising approximately 4100 species distributed across 124 genera, is particularly diverse in Mediterranean regions [4]. Several Ericaceae species are traditionally used for the treatment of various disorders, notably those related to urinary tract infections and wound healing [2]. Their biological effects are mainly attributed to phenolic compounds and other secondary metabolites with promising pharmacological properties [5]. Within this family, the genus *Erica* stands out for its wide geographical distribution and remarkable diversity [6], comprising more than 800 species, predominantly evergreen shrubs adapted to nutrient‐poor soils [7]. The genus exhibits substantial morphological and physiological diversity, making it an excellent model for studies in evolutionary biology, ecology, and plant physiology [8]. Moreover, *Erica* species have long been used in traditional medicine for their anti‐ulcer, anti‐inflammatory, antinociceptive, antimicrobial, antilithiasic, cytotoxic, and antioxidant properties [3]. Among these species, *Erica arborea* L., commonly known as tree heath, is an evergreen shrub or small tree reaching 1–4 m in height [9]. It is widely distributed throughout the Mediterranean region, as well as in western Portugal, the Canary Islands, and North Africa, particularly in Morocco, Tunisia, and Algeria [1]. In traditional medicine, *E. arborea* is extensively used for the treatment of various ailments. Its leaves and flowers are mainly employed for their diuretic, astringent, antirheumatic, and urinary antiseptic properties, contributing to the management of urinary tract disorders, including infections [10], and constipation [5]. In addition, the aerial parts of the plant are recognized for their anti‐inflammatory [11], anti‐ulcer, antibacterial, antidiarrheal, anti‐edematous, cytotoxic, and wound‐healing activities [1]. Traditional preparations also describe the external use of aerial parts for the treatment of wounds, snake bites [9], and insect stings. In northern Algeria, the flowers of *E. arborea* are widely recommended for the treatment of enuresis and kidney stones, commonly administered as infusions or decoctions [2], while its wood (briar root) is traditionally used in the manufacture of smoking pipes and jewellery [10]. From a phytochemical perspective, *E. arborea* contains a wide range of secondary metabolites, including flavonoids, phenolic compounds [11], alkaloids [6], monoterpenes, triterpenoids, phenylpropanoid glycosides, and condensed tannins. Several studies have demonstrated the biological activities of its extracts, including antioxidant, anti‐inflammatory, analgesic, and antinociceptive effects [9]. However, despite the available data, further investigations are needed to better characterize the chemical composition of *E. arborea* and clarify the relationship between its phytochemical constituents and biological activities, particularly through combined analytical and computational approaches. In this context, the present study aims to investigate the biological potential of *E. arborea* collected in Algeria using an integrated approach. The antimicrobial and antioxidant activities of its organic extracts were evaluated, followed by their chemical characterization to identify the major phytoconstituents. Furthermore, molecular docking analyses were performed to explore the potential interactions between these major compounds and key antioxidant and antibacterial targets, providing mechanistic insights into the experimentally observed biological activities.

## 2 Materials and Methods

### 2.1 Plant Collection and Secondary Metabolite Extraction

The nonflowering aerial parts of *E. arborea* were collected in November 2024 from the Draa en Naga forest in Constantine, Algeria (36°22′26.2″N, 6°44′02.0″E). The plant was identified by Prof. Hocine Laouer (University Ferhat Abbas, Sétif 1, Algeria), and a voucher specimen (No. ERA2024) was deposited in the VARENBIOMOL Unit, University of Mentouri Constantine 1. The plant material was carefully washed and dried in a well‐ventilated, light‐protected area before being finely ground into powder. Secondary metabolites were extracted following the protocol described by Bramki et al. [12]. Briefly, 20 g of plant powder were macerated in 300 mL of a methanol/water mixture (1:1 v/v) at room temperature under continuous agitation for 48 h. The resulting mixture was filtered through Whatman No. 1 filter paper to remove plant debris. The solvent was evaporated under reduced pressure using a rotary evaporator. The aqueous phase was subsequently extracted sequentially with *n‐*hexane, dichloromethane, and ethyl acetate (3 × 300 mL each), and the resulting fractions were concentrated under reduced pressure. The extraction yield was calculated on a dry weight basis using the following formula [13]:

$$Y\left(\%\right)=\frac{\text{weight of dried extract collected}}{\text{weight of dried plant material}}\times 100$$

### 2.2 Determination of Total Phenolic, Total Flavonoids, and Flavonols Contents

#### 2.2.1 Determination of Total Phenolic Content (TPC)

The TPC of the three extracts was assessed using the Folin Ciocalteu reagent (FCR) method [14]. In brief, 20 µL of each extract was mixed with 80 µL of 7.5% (w/v) sodium carbonate and 100 µL of FCR (previously diluted 1:10 with deionized water). The mixture was incubated in the dark for 2 h, and the absorbance was then measured at 765 nm using a microplate reader (Perkin Elmer, EnSpire). Gallic acid was employed as the standard to generate a calibration curve (*Y* = 0.0034*X* + 0.2205, *R* ^2^ = 0.9624), and TPC was expressed as µg of gallic acid equivalents per mg of dry extract (µg GAE/mg).

#### 2.2.2 Determination of Total Flavonoids Content (TFC)

The TFC of each extract was evaluated using Topçu et al. [15]. method, with slight modifications. Briefly, 50 µL of extract was combined with 130 μL of methanol, 10 µL of potassium acetate 9.8% (w/v), and 10 µL of aluminum nitrate solution (10%). The reaction mixture was allowed to develop for 40 min at room temperature, and the absorbance was subsequently recorded at 415 nm. Methanol was used as a blank, and the TFC was determined using a calibration curve constructed with quercetin (*Y* = 0.0085*X* + 0.04710, *R* ^2^ = 0.9954). The results were expressed as µg of quercetin equivalents per mg of dry extract (µg QE/mg).

#### 2.2.3 Determination of Total Flavonols Content (TFolC)

The TFolC of extracts was determined following the method described by Kumaran and Joel Karunakaran [16]. In brief, 50 µL of each extract was mixed with 50 µL of aluminum trichloride (2%) and 150 µL of sodium acetate (5%). The reaction mixture was left to incubate in the dark for 90 min, and the absorbance was recorded at 440 nm. Methanol was used as a blank, and a quercetin standard curve (*Y* = 0.0109*X* + 0.1081, *R* ^2^ = 0.9979) was employed to calculate the TFolC, which was expressed as µg of quercetin equivalents per mg of dry extract (µg QE/mg).

### 2.3 GC–MS Analysis

GC–MS data were acquired on the raw crude extracts (i.e., *n*‐hexane, dichloromethane, and EtOAc extracts) of *E. arborea* with *N*,*O*‐bis(trimethylsilyl)trifluoroacetamide (BSTFA) (Fluka, Buchs, Switzerland). In particular, 100 μL of BSTFA and 100 μL of acetonitrile were added to each sample and incubated for 2 min in a microwave oven, as previously described [17]. Trimethylsilyl derivatives of metabolites were analyzed using an Agilent 6850 GC (Milan, Italy), equipped with an HP‐5 MS capillary column (stationary phase: (5%‐phenyl)‐methylpolysiloxane; length: 30 m; ID: 0.25 mm; film thickness: 0.25 µm), coupled to an Agilent 5973 Inert MS detector operated in the full scan mode (*m/z* 40–550) at a frequency of 3.9 Hz and with the EI ion source and quadrupole mass filter temperatures kept, respectively, at 200 and 250 °C. Helium was used as carrier gas at a flow rate of 1 mL/min. The injector temperature was 250 °C, and the temperature ramp raised the column temperature from 70 to 280 °C: 70 °C for 1 min; 10 °C/min until reaching 170 °C; and 30 °C for 1 min until reaching 280 °C. Then, it was held at 280 °C for 5 min. The solvent delay was 4 min. Metabolites were identified by comparing their EI mass spectra at 70 eV with mass spectra collected in the NIST 20 mass spectral library (available online: https://www.nist.gov/srd/nist‐standard‐reference‐database‐1a (accessed on 15 December 2025). Moreover, the identification was supported by the Kovats retention index (RI) calculated for each metabolite by the Kovats equation, using the standard *n*‐alkane mixture in the range C7–C40.

The chromatographic peak area percentage of each identified compound was calculated using the following formula:

$$\text{Peak area percentage}=\frac{\text{Individual peak area}}{\text{Total area of all peaks}}\times 100$$

### 2.4 Antioxidant Ability

The antioxidant activity of the extracts was assessed using four complementary approaches: the DPPH^•^ (2,2‐diphenyl‐1‐picrylhydrazyl) and ABTS^•+^ ((2,2′‐azino‐bis(3‐ethylbenzothiazoline‐6‐sulfonic acid) free radical‐scavenging assays, as well as the FRAP (Ferric Reducing Antioxidant Power) and phenanthroline tests. The assays were performed in 96‐well microplates. Trolox and ascorbic acid served as antioxidant standards.

#### 2.4.1 DPPH^•^ Scavenging Assay

The test was assessed following the protocol established by Tel et al. [18]. For this assay, a 0.1 mM DPPH^•^ solution was prepared using methanol as the solvent. Then, 160 μL of this solution was mixed with 40 μL of each extract, previously diluted in methanol at different concentrations. After a 15 min incubation period, the absorbance of the mixture was measured at 517 nm. The results are expressed as IC_50_ values (μg/mL). The DPPH^•^ radical scavenging capacity was calculated using the following equation:

$$\text{DPPH scavenging effect}\left(\%\right)=\frac{{\text{Absorbance}}_{\text{Control}}-{\text{Absorbance}}_{\text{sample}}}{{\text{Absorbance}}_{\text{Control}}}\times 100$$

#### 2.4.2 ABTS^•+^ Assay

For the ABTS^•+^ assay, 40 μL of each extract, previously prepared in methanol at varying concentrations, was mixed with 160 μL of the ABTS^•+^ solution. The reaction mixture was kept in the dark for 10 min, after which its absorbance was measured at 734 nm. A mixture of methanol and the ABTS^•+^ solution was used as the blank. The percentage of radical scavenging was determined using the same calculation formula applied in the DPPH^•^ assay [19].

#### 2.4.3 FRAP Assay

Following the method described by Oyaizu [20], a volume of 10 μL of each extract at various concentrations was mixed with 40 μL of phosphate buffer (0.2 M, pH 6.6) and 50 μL of potassium ferricyanide (1%). The mixture was incubated at 50 °C for 20 min, after which the reaction was stopped by adding 50 μL of trichloroacetic acid (10%). Subsequently, 40 μL of distilled water and 10 μL of ferric chloride (FeCl_3_, 0.1%) were added. Methanol was used as the blank during measurement, and the absorbance was then recorded at 700 nm to determine the reducing power. The results were expressed as A_0.50_ (μg/mL), corresponding to the concentration required to reach an absorbance of 0.50.

#### 2.4.4 Phenanthroline Assay

According to the procedure described by Szydłowska‐Czerniak [21], a volume of 10 μL of each extract was combined with 50 μL of FeCl_3_ (0.2%), 30 μL of phenanthroline (0.5%), and 110 μL of methanol. The mixture was then incubated in the dark at 30 °C for 20 min to allow the Fe^2+^–phenanthroline complex to develop. The absorbance of the resulting red–orange complex was recorded at 510 nm using a spectrophotometer. The results were expressed as A_0.50_ (μg/mL).

### 2.5 Antimicrobial Activity Assay

The antimicrobial potential of the three extracts was evaluated using the agar well diffusion method against a panel of reference microorganisms. The bacterial strains tested were *Staphylococcus aureus* (ATCC 25923), *Bacillus subtilis* (ATCC 6633), *Escherichia coli* (ATCC 25922), *Pseudomonas aeruginosa* (ATCC 27853), and *Salmonella typhimurium* (ATCC 14028). The antifungal activity was assessed against five fungal strains, including *Aspergillus niger* (MH109542), *Aspergillus fumigatus* (MH109539), *Fusarium oxysporum*, *Penicillium* sp., and *Candida albicans*.

Bacterial strains were reactivated by subculturing on appropriate selective media and incubated at 37 °C for 18 h. Fresh bacterial suspensions were prepared in sterile physiological saline [22]. Similarly, *C. albicans* was cultured on Sabouraud agar at 37 °C for 24 h, and the yeast suspension was prepared in sterile physiological water. In both cases, the turbidity of the microbial suspensions was adjusted to the 0.5 McFarland standard [23]. Filamentous fungi were subcultured on Potato Dextrose Agar (PDA) and incubated at 28 °C for 14 days. Spore suspensions were obtained by scraping the surface of the cultures after the addition of sterile physiological water. The suspensions were adjusted to an absorbance between 0.15 and 0.20 at 650 nm, then diluted to 1/10^th^ [24]. The antibacterial activity was evaluated on Mueller–Hinton agar plates previously inoculated with standardized bacterial suspensions by surface spreading. Wells of 6 mm diameter were aseptically punched into the agar. Each well was filled with 40 µL of the extract solution (50 mg/mL prepared in dimethyl sulfoxide, DMSO). The plates were kept at 4 °C for 30 min to allow diffusion of the extracts, then incubated at 37 °C for 24 h. Gentamicin (10 µg/disc) was used as a positive control, while DMSO served as a negative control [25]. For antifungal activity, the assay was carried out on Sabouraud dextrose agar following the same well diffusion procedure. Plates inoculated with *C. albicans* were incubated at 37 °C for 24–48 h, whereas those inoculated with filamentous fungi were incubated at 28 °C for 48–72 h. Nystatin (5 mg/mL) was used as the positive control.

Antimicrobial activity was assessed by measuring the diameter of the inhibition zones surrounding the wells. Results were expressed in millimeters (mm), including the diameter of the well [26, 27].

### 2.6 Statistical Analysis

All experiments were carried out in triplicate, and the results are expressed as mean values ± standard deviation (SD). The effects of solvent type on the biological activities were evaluated using a one‐way analysis of variance (ANOVA), followed by Tukey's HSD post‐hoc test for pairwise comparisons. Statistical analyses were performed using XLSTAT software (Addinsoft SARL, New York, NY, USA). Differences were considered statistically significant at *p* ≤ 0.05.

### 2.7 Molecular Docking

Molecular docking analyses were performed to explore the possible binding interactions between the main phytoconstituents of the *E. arborea* EtOAc extract and selected antioxidant and antibacterial protein targets. All computational procedures were conducted using UCSF Chimera v1.16 for protein and ligand preparation, AutoDock Vina v1.2.7 for docking simulations, and Discovery Studio Visualizer v2.1.1 for visualization and interaction analysis. The chemical structures of all compounds identified in the extract were retrieved from PubChem in SDF format, while the crystallographic structures of the selected proteins were downloaded from the RCSB Protein Data Bank (PDB).

The selected targets included xanthine oxidase (XO, PDB ID: 1N5X) to investigate the antioxidant activity. The XO structure selected for this study (PDB ID: 1N5X) corresponds to bovine xanthine oxidase (*Bos taurus*), a crystallographic model extensively used for inhibitor screening due to its well‐resolved active site and the availability of a co‐crystallized inhibitor (TEI‐6720). In addition, bovine and human XO share a highly conserved catalytic pocket and similar enzymatic functions, making bovine XO a reliable surrogate model for the investigation of potential XO inhibitors. XO was selected as the antioxidant target because it is a major source of reactive oxygen species (ROS) during purine metabolism, and its inhibition is recognized as an important mechanism underlying antioxidant activity. Furthermore, the availability of a high‐quality crystal structure with a bound inhibitor enabled reliable docking protocol validation through redocking experiments.

The antibacterial potential was investigated using two bacterial DNA gyrase enzymes, *B. subtilis* GyrA (PDB ID: 4DDQ), and *S. aureus* GyrB (PDB ID: 3G75), to explore the antibacterial potential. Protein preparation was carried out in UCSF Chimera by removing crystallographic water molecules and co‐crystallized ligands (which were retained separately for redocking validation), adding hydrogens with appropriate protonation states at physiological pH, and assigning Gasteiger charges. Proteins were then subjected to a brief energy minimization using the AMBER ff14SB force field to relieve steric clashes and optimize side‐chain and hydrogen orientations.

Ligand structures were imported from PubChem, converted into PDBQT format, and geometry‐optimized in UCSF Chimera. Gasteiger charges were assigned, and rotatable bonds were automatically detected [28]. Reference ligands used for validation included TEI‐6720 for XO, ciprofloxacin for GyrA, and the co‐crystallized ligand B48 for GyrB.

The reliability of the docking protocol was assessed through a redocking procedure. For each protein, the co‐crystallized ligand was extracted from the active site, prepared following the same steps applied to the test ligands, and re‐docked into the native binding pocket using AutoDock Vina with identical grid parameters. The docked conformations were aligned with the crystallographic poses, and the root‐mean‐square deviation (RMSD) was calculated in UCSF Chimera. All simulations yielded RMSD values below 1.7 Å, confirming the accuracy and robustness of the docking protocol and validating its suitability for subsequent ligand screening [29].

Docking simulations were then performed for all selected metabolites by centering the grid box on the position of the co‐crystallized ligand of each target to ensure precise coverage of the active site. The best pose for each compound was selected based on the lowest binding energy. In accordance with the in vitro findings, only ligands exhibiting binding energies equal to or stronger than the reference inhibitors for each target were retained for interpretation, ensuring a focused analysis on the most biologically relevant compounds.

Protein–ligand complexes were finally examined using Discovery Studio Visualizer to identify key molecular interactions, including hydrogen bonds, hydrophobic contacts (alkyl, π–alkyl), π–π stacking, π–cation and π–anion interactions, and salt bridges. Two‐dimensional interaction diagrams and 3D structural representations were generated to support the interpretation of the docking outcomes.

## 3 Results and Discussion

To better understand the relationship between phytochemical composition and biological activity, the crude hydroalcoholic extract of *E. arborea* was sequentially partitioned using solvents of increasing polarity, namely *n*‐hexane, dichloromethane, and EtOAc. This strategy was designed to selectively concentrate different classes of secondary metabolites according to their polarity, thereby facilitating the identification of the fractions responsible for the observed biological activities. *n*‐Hexane, a nonpolar solvent, preferentially extracts lipophilic constituents such as fatty acids, hydrocarbons, terpenoids, and sterols, whereas dichloromethane, with intermediate polarity, is more suitable for recovering moderately lipophilic metabolites, including terpenoids, sterols, alkaloids, and certain phenolic derivatives. In contrast, EtOAc efficiently extracts moderately polar compounds, particularly phenolic acids and flavonoid aglycones, which are widely recognized for their antioxidant and antimicrobial activities. Therefore, sequential solvent partitioning not only improves phytochemical discrimination among the fractions but also facilitates the correlation between the chemical composition of each fraction and its biological properties, allowing the identification of the most promising source of bioactive compounds [30, 31, 32].

The extraction yields of the three solvent fractions were determined to compare the extraction efficiency of the different solvents and are presented in Table 1.

**TABLE 1:** Extraction yields of the three *E. arborea* organic extracts.

	*n‐*hexane	Dichloromethane	EtOAc
Extraction yield, %	1.40	1.88	5.58

The results indicate that the yield strongly depends on the solvent used, reflecting the specific affinity of each solvent for the secondary metabolites present in *E. arborea*. EtOAc proved to be the most effective, with a maximum yield of 5.58%, highlighting its ability to extract a wide range of polar compounds, consistent with the findings of Ay et al. [33]. These results also corroborate the data reported by Amari et al. [6], who obtained yields of 5.32% for flowers and 6.84% for leaves, confirming the efficiency of this solvent in extracting polar compounds. These observations suggest that the majority of metabolites in *E. arborea* are polar compounds. In contrast, the yield obtained with *n*‐hexane was low (1.4%), indicating a low content of nonpolar compounds in the plant. Dichloromethane extraction gave an intermediate yield of 1.88%, similar to the values reported by Amari et al. [6] for chloroform (1.2% for flowers and 1.36% for leaves). Since these solvents are generally used to isolate moderately nonpolar compounds, these results suggest that this type of metabolite is relatively scarce in *E. arborea*.

### 3.1 Determination of TPC, TFC, and TFolC

The TPC, TFC, and TFolC were determined using spectrophotometric methods. TPC was quantified using the Folin–Ciocalteu reagent, with gallic acid as the reference standard, and the results were expressed as μg gallic acid equivalents per mg of extract (μg GAE/mg extract). TFC and TFolC were quantified by the aluminum chloride (AlCl_3_) colorimetric method, using quercetin as the standard, and the results were expressed as μg quercetin equivalents per mg of extract (μg QE/mg extract) (Table 2).

**TABLE 2:** TPC, TFC, and TFolC values of the three *E. arborea* fractions (*n* = 3).

Extract	TPC, µg GAE/mg	TFC, µg QE/mg	TFolC, µg QE/mg
*n‐*hexane	8.35 ± 1.83^c^	8.28 ± 0.73^c^	14.11 ± 1.75^c^
Dichloromethane	48.70 ± 1.34^b^	26.22 ± 1.53^b^	45.49 ± 1.61^b^
EtOAc	419.58 ± 1.89^a^	174.57 ± 1.93^a^	61.64 ± 2.49^a^

*Note*: Values are mean ± standard error (SE) of three replicates. Different letters within the same column indicate significant differences among treatments according to Tukey's HSD test (*p* ≤ 0.05).

Among the three extracts investigated, the EtOAc extract exhibited the highest levels of TPC, TFC, and TFolC, with contents of 419.58 ± 1.89 μg GAE/mg extract, 174.57 ± 1.93, and 61.64 ± 2.49 μg QE/mg extract, respectively, followed by the dichloromethane and *n*‐hexane fractions, with significant differences observed among all fractions (*p* ≤ 0.05). These results highlight the superior ability of EtOAc to concentrate phenolic constituents, which can be attributed to its intermediate polarity and strong affinity for phenolic acids and flavonoids. This observation is consistent with the recent review by Benmanseur et al. [34] which emphasized that solvent polarity is a key factor influencing the extraction efficiency of phenolic compounds in *Erica* species, with moderately polar solvents generally yielding phenolic‐rich extracts associated with enhanced biological activities. Nevertheless, quantitative variations in phenolic content among studies are expected because they are influenced by several factors, including plant species, geographical origin, plant material, phenological stage, environmental conditions, extraction methods, and solvent polarity [35]. In the present study, the TPC of the EtOAc extract was lower than that reported by Köroğlu et al. [3]. In their work, the authors investigated the aerial parts of four *Erica* species (*E. arborea*, *E. bocquetii*, *E. manipuliflora*, and *E. sicula* subsp. *libanotica*) using solvents of different polarities, including water, methanol, chloroform, EtOAc, and *n*‐butanol. They reported TPC ranging from 44.9 to 875.5 mg GAE/g extract, with the highest value obtained from the EtOAc extract of *E. arborea*. The other species also exhibited relatively high TPC with this solvent, although at levels lower than those observed for *E. arborea*. Similarly, Yaici et al. [36] analyzed the aqueous extracts of *E. arborea* leaves and flowers, reporting TPC of 74.22 ± 0.01 and 60.88 ± 0.02 mg GAE/g dry matter, respectively. Comparable results were reported by Amezouar et al. [4], who analyzed the leaf extract of *E. arborea* and found a TPC of 78.49 ± 0.047 mg GAE/g extract. Regarding TFC, the EtOAc extract in our study also exhibited the highest levels, exceeding the values reported by Yaici et al. [36], who found 55.54 ± 0.47 mg QE/g extract in leaves and 28.18 ± 0.31 mg QE/g extract in flowers from aqueous extracts, as well as those reported by Amezouar et al. [4] for the leaf extract, which was 54.08 ± 0.031 mg QE/g. Furthermore, Amari et al. [6] confirmed the superiority of EtOAc, reporting TFC values of 67.15 ± 0.04 μg QE/mg in leaves and 65.31 ± 0.56 μg QE/mg in flowers. Other extracts, including methanol, chloroform, and aqueous extracts, showed lower values ranging from 6.02 ± 0.11 to 51.12 ± 1.42 μg QE/mg, highlighting the particular affinity of this solvent for these compounds. Indeed, flavonols were also predominantly extracted with EtOAc, indicating that this solvent is the most efficient for concentrating flavonols as well. To our knowledge, no previous studies have quantified flavonols in *E. arborea* extracts, or more broadly within the genus *Erica*.

### 3.2 GC–MS

*n*‐Hexane, dichloromethane, and EtOAc extracts of *E. arborea* have been analyzed via GC–MS after derivatization with BSTFA, as reported in the Material and Methods Section. As can be seen from Table 3, our data showed that the extracts are rich in diverse low‐molecular‐weight compounds. At first glance, it is evident that there are numerous fatty acids in the crude extracts, including palmitic acid, myristic acid, and stearic acid. GC–MS analysis also revealed the presence of glycerol esters with palmitic and stearic acids. Several phenolic compounds and their derivatives (e.g., protocatechuic acid, methyl caffeate, and quininic acid) have been detected, especially in the crude extracts obtained with dichloromethane and EtOAc. Furthermore, catechine, a well‐known flavonoid, has been detected in the EtOAc extract.

**TABLE 3:** Chromatographic peak area percentages of compounds identified via GC–MS in crude extracts (i.e., *n*‐hexane, dichloromethane, and EtOAc extracts) of *Erica arborea* after trimethylsilylation with BSTFA.

Compound	Class of compounds	RI	*n*‐hexane extract, %	Dichloromethane extract, %	EtOAc extract, %
Lactic Acid, 2TMS	Hydroxy acids	1051	8.55	3.47	2.08
Urea, 2TMS	Diamides	1246	0.57		1.01
Benzoic Acid, TMS	Aromatic carboxylic acids	1254		1.34	
Glycerol, 3TMS	Polyalchols	1280	2.67	2.78	1.07
Phosphate, 3TMS	Inorganic compounds	1283	0.28	2.44	1.24
Nonanoic acid, TMS	Fatty acids	1357	0.68		
Decanoic acid, TMS	Fatty acids	1454	0.40		
2,4‐Di‐tert‐butylphenoxytrimethylsilane	Phenols	1549	2.06		
Dodecanoic acid, TMS	Fatty acids	1648	0.52		6.89
Protocatechuic acid, 3TMS	Phenolic acids	1823			1.76
Myristic acid, TMS	Fatty acids	1841	1.77		
Quininic acid, 5TMS	Quinolines	1881	4.45		2.85
Methyl caffeate, 2TMS	Phenylpropanoids	2015		3.78	
Palmitic Acid, TMS	Fatty acids	2040	19.09	7.40	6.96
Stearic acid, TMS	Fatty acids	2230	3.84		3.19
Oleamide, TMS	Fatty amides			27.18	
1‐Monopalmitin, 2TMS	Monoglycerols	2595	31.87	24.10	4.65
Glycerol monostearate, 2TMS	Monoglycerols	2793	23.24	27.51	54.92
Catechine, 5TMS	Flavanols	2884			13.38

Abbreviations: RI, Kovats retention index; TMS, trimethylsilyl group.

A complementary relationship was observed between the spectrophotometric assays and the GC–MS analysis. The spectrophotometric assays provide an overall estimation of TPC, TFC, and TFolC, whereas GC–MS identifies individual low‐molecular‐weight metabolites after derivatization. Therefore, the results obtained by these approaches are complementary rather than directly comparable. The EtOAc fraction, which exhibited the highest TPC, TFC, and TFolC values, also contained phenolic metabolites such as protocatechuic acid and catechin, supporting the enrichment of this fraction in phenolic constituents. In contrast, the dichloromethane fraction displayed intermediate spectrophotometric values and was characterized by the presence of methyl caffeate, suggesting a lower abundance of phenolic constituents than in the EtOAc fraction. The *n*‐hexane fraction showed the lowest TPC, TFC, and TFolC values, which is consistent with its GC–MS profile dominated by fatty acids, monoacylglycerols, and other lipophilic compounds that do not contribute significantly to the colorimetric determination of phenolics. It should also be emphasized that GC–MS mainly detects volatile or derivatizable metabolites. Consequently, high‐molecular‐weight polyphenols, glycosylated flavonoids, tannins, and other nonvolatile phenolic compounds that contribute to the spectrophotometric assays may not be detected by this technique. Thus, combining spectrophotometric assays with GC–MS provides a more comprehensive characterization of the phytochemical composition of *E. arborea* extracts.

### 3.3 Antioxidant Ability

The in vitro antioxidant activity of *E. arborea* extracts obtained using solvents of increasing polarity (*n*‐hexane, dichloromethane, and EtOAc) was evaluated using DPPH^•^, ABTS^•+^, phenanthroline, and FRAP assays. The results demonstrated a strong dependence of antioxidant capacity on the extraction solvent and the assay type (Table 4).

**TABLE 4:** In vitro antioxidant activity of *E. arborea* via DPPH^•^, ABTS^•+^, phenanthroline, and FRAP assays.

	IC_50_, µg/mL		A_0.5_, µg/mL	
	DPPH^•^	ABTS^•+^	Phenanthroline	FRAP
*n‐*Hexane	>800^a^	792.42 ± 3.63^a^	6.72 ± 0.15^a^	>200^a^
Dichloromethane	346.07 ± 1.80^b^	266.08 ± 0.97^b^	6.49 ± 0.15^a^	141.34 ± 1.52^b^
EtOAc	26.96 ± 1.16^c^	5.07 ± 0.63^c^	1.25 ± 0.05^d^	18.14 ± 0.57^c^
Trolox	5.14 ± 0.15^d^	3.27 ± 0.30^c^	5.21 ± 0.11^b^	5.43 ± 0.53^d^
Ascorbic acid	4.40 ± 0.18^d^	3.07 ± 0.07^c^	3.08 ± 0.08^c^	3.76 ± 0.39^d^

*Note:* Values are mean ± standard error (SE) of three replicates. Different letters within the same column indicate significant differences among treatments according to Tukey's HSD test (*p* ≤ 0.05).

Overall, the EtOAc fraction showed the highest antioxidant activity across all assays. Significant differences among extracts were confirmed by one‐way ANOVA (*p* ≤ 0.05). The EtOAc extract exhibited markedly lower IC_50_ values in the DPPH^•^ and ABTS^•**+**^ assays and the strongest reducing power in both the phenanthroline and FRAP assays. In the phenanthroline assay, all extracts demonstrated the greatest reducing capacity, indicating their ability to donate electrons, although with varying intensities. The EtOAc fraction displayed the most pronounced effect, while both dichloromethane and *n*‐hexane extracts showed marked but lower reducing activities, suggesting the presence of redox‐active compounds even in the less polar fractions. This pattern contrasts with the DPPH^**•**^, ABTS^•**+**^, and FRAP assays, in which the dichloromethane extract exhibited moderate activity, and *n*‐hexane extract exhibited very weak activity. The antioxidant performance of the EtOAc fraction was comparable to that of the reference antioxidants Trolox and ascorbic acid, particularly in the ABTS^•**+**^ assay, where no statistically significant difference was observed. These findings indicate that solvent polarity plays a critical role in the extraction of antioxidant constituents, with semi‐polar solvents such as EtOAc being most effective for isolating bioactive compounds from *E. arborea*. The observed antioxidant profile aligns with previous studies on *E. arborea* and related *Erica* species, which consistently reported higher antioxidant activities in polar or semi‐polar extracts compared to nonpolar ones. For instance, a recent study by Amari et al. [6] on *E. arborea* leaves and flowers showed that the EtOAc subfractions possessed the highest total phenolic and flavonoid contents, and exhibited strong DPPH^**•**^ radical scavenging (IC_50_ ≈ 17.7 µg/mL) and reducing power activity. Similarly, an investigation on stem extracts of *E. arborea* confirmed that the EtOAc fraction displayed the strongest antioxidant activity in DPPH^**•**^ (IC_50_ = 0.02 mg/mL) assay [37]. Moreover, Amezouar et al. [4] reported potent DPPH^**•**^ scavenging in Moroccan *E. arborea* ethanolic leaf extracts (IC_50_ ≈ 10.22 µg/mL). Additionally, Zengin et al. [5] demonstrated that leaf extracts of *E. arborea* exhibit marked antioxidant properties, as evidenced by multiple assays, including DPPH^**•**^, ABTS^•**+**^, CUPRAC, FRAP, and metal chelating tests, reflecting both radical scavenging and reducing capacities. Collectively, these findings indicate that *E. arborea* extracts act as effective electron donors and metal ion chelators, supporting their role in mitigating oxidative stress through complementary antioxidant mechanisms. The antioxidant potential of *E. arborea* extracts can be directly linked to their chemical composition as revealed by GC–MS analysis (Table 3). The EtOAc fraction, which exhibited the highest antioxidant capacity, contained several bioactive compounds known for their redox properties, including catechin**,** protocatechuic acid, and quinic acid, in addition to organic acids and fatty acids. In particular, the detection of catechin, a flavonoid with multiple hydroxyl groups, strongly supports the high antioxidant activity observed in the EtOAc extract, as this compound is known for its powerful electron‐donating capacity [38, 39]. Quinic acid, also identified in this fraction, is reported to exert moderate antioxidant effects and may synergistically enhance the activity of phenolic compounds [40]. The combination of these compounds with fatty acids likely contributes to the superior antioxidant performance of the EtOAc fraction, in agreement with previous studies showing that extracts containing mixtures of flavonoids, phenolic acids, and fatty acids exhibit stronger activity than extracts dominated by a single class of compounds, due to synergistic interactions that enhance radical scavenging and reducing capacity [41, 42]. The dichloromethane fraction, which showed moderate antioxidant activity, contained a mixture of lipophilic compounds such as oleamide, palmitic acid, and glycerol esters, along with the phenolic derivative methyl caffeate. The presence of methyl caffeate, a phenolic ester with known antioxidant activity, likely contributes to the free‐radical scavenging ability of this extract [43]. However, the predominance of nonphenolic lipophilic components may limit the overall antioxidant capacity compared to the polyphenol‐rich EtOAc extract. In contrast, the *n*‐hexane extract exhibited the weakest activity and was mainly composed of long‐chain fatty acids, including palmitic, stearic, and myristic acids, along with glycerides and other nonphenolic constituents. Although fatty acids such as palmitic acid and stearic acid have been reported to exhibit mild antioxidant effects [44], their activity remains considerably weaker compared to that of flavonoids and phenolic acids [45]. Furthermore, the presence of 2,4‐di‐tert‐butylphenol, a known antioxidant compound, may partially contribute to the measured activity [46, 47]; however, its relatively low abundance compared to the dominant nonpolar constituents likely limits its overall impact. Collectively, these findings demonstrate a clear relationship between the chemical composition of *E. arborea* extracts and their antioxidant capacity, highlighting phenolic compounds as the primary contributors to the observed activity and confirming the critical role of solvent polarity in extracting bioactive constituents.

### 3.4 Antimicrobial Activity

The antibacterial activity of *E. arborea* fractions was evaluated against selected Gram‐positive (*S. aureus* and *B. subtilis*) and Gram‐negative bacteria (*E. coli*, *P. aeruginosa*, and *S. typhimurium*) using the agar diffusion method. The results, presented in Table 5, indicate that *E. arborea* extracts exhibited varied antibacterial activity, with significant differences among the fractions tested.

**TABLE 5:** In vitro antibacterial activity of *E. arborea* fractions.

	Inhibition Zones, mm				
	*S. aureus*	*B. subtilis*	*E. coli*	*P. aeruginosa*	*S. typhimurium*
*n‐*Hexane	—	—	—	—	—
Dichloromethane	10.33 ± 0.57^b^	9.33 ± 0.57^b^	—	—	—
EtOAc	19.33 ± 0.57^a^	19.0 ± 1.0^a^	—	—	—
Gentamicin	18.0 ± 0.5^a^	19.1 ± 0.8^a^	16.2 ± 0.9	17.17 ± 0.25	15.5 ± 0.7

*Note:* —: Inactive. Values are mean ± standard error (SE) of three replicates. Different letters within the same column indicate significant differences among treatments according to Tukey's HSD test (*p* ≤ 0.05). For DMSO, no antimicrobial activity was observed against any of the tested strains.

The *n*‐hexane fraction showed no antibacterial effect against any of the tested strains, whereas the dichloromethane fraction displayed moderate antibacterial activity against Gram‐positive species, with inhibition zones of 9.33–10.33 mm. These values were significantly lower than those observed for the gentamicin control, which had inhibition zones of 18.0 mm against *S. aureus* and 19.1 mm against *B. subtilis*. The EtOAc fraction exhibited the strongest antibacterial activity, producing inhibition zones of 19.33 and 19.0 mm for *S. aureus* and *B. subtilis*, respectively, which were statistically comparable to or even slightly more effective than gentamicin, particularly against *B. subtilis*. Statistical analysis revealed significant differences (*p* ≤ 0.05) between fractions, with the EtOAc fraction being significantly more effective than the dichloromethane and *n*‐hexane fractions; however, none of the fractions showed inhibitory activity against Gram‐negative bacteria. In addition to antibacterial activity, *E. arborea* fractions were tested for antifungal activity against several fungal strains (*C. albicans*, *A. niger*, *A. fumigatus*, *Penicillium* sp., and *F. oxysporum*), as presented in Table 6. The antifungal potential of the *E. arborea* fractions varied significantly between fungal strains.

**TABLE 6:** Antifungal activity of *E. arborea* fractions.

	Inhibition zones, mm				
	*C. albicans*	*A. niger*	*A. fumigatus*	*Penicillium* sp.	*F. oxysporum*
*n‐*Hexane	8.0 ± 0.0^d^	—	—	—	10.0 ± 1.0^c^
Dichloromethane	10.66 ± 0.57^c^	—	—	—	13.33 ± 1.15^b^
EtOAc	13.66 ± 1.15^b^	—	—	—	15.66 ± 1.15^b^
Nystatin	31.5 ± 1.5^a^	35.0 ± 0.9	36.8 ± 1.2	32.5 ± 0.6	30.5 ± 0.3^a^

*Note:* —: Inactive. Values are mean ± standard error (SE) of three replicates. Different letters within the same column indicate significant differences among treatments according to Tukey's HSD test (*p* ≤ 0.05). For DMSO, no antimicrobial activity was observed against any of the tested strains.

All fractions showed antifungal activity against *C. albicans* and *F. oxysporum*, while no inhibition was observed against other filamentous fungi, indicating a selective antifungal effect. Again, activity increased significantly with solvent polarity; *n*‐hexane fraction demonstrated limited antifungal activity, with an inhibition zone of 8.0 mm against *C. albicans* and 10.0 mm against *F. oxysporum*. The dichloromethane fraction showed slightly improved activity, with inhibition zones of 10.66 mm against *C. albicans* and 13.33 mm against *F. oxysporum*, whereas the EtOAc fraction exhibited the strongest antifungal effects, producing inhibition zones of 13.66 mm against *C. albicans* and 15.66 mm against *F. oxysporum*. These values, while significantly lower than the nystatin control (*p* ≤ 0.05), indicate promising antifungal properties. The antimicrobial activity observed in our study is broadly consistent with earlier research on *E. arborea*. Amari et al. [37] reported moderate antibacterial activity of methanolic and subfractions of *E. arborea* stems, although no antifungal activity was detected. Similarly, Amari et al. [2] found that hydromethanolic flower extracts inhibited Gram‐positive bacteria and, to a lesser extent, certain Gram‐negative species. This aligns with the findings of Guendouze‐Bouchefa et al. [1], who reported that methanolic extracts from the flowering aerial parts of *E. arborea* exhibited significant activity against Gram‐positive strains such as *S. aureus* ATCC 6538 and *S. aureus* C100459 (methicillin‐resistant), while no activity was observed against Gram‐negative bacteria, including *P. aeruginosa* and *E. coli*. Furthermore, Yaici et al. [36] studied aqueous extracts of *E. arborea* leaves and flowers. Their study revealed no activity against *E. coli*, *Streptococcus* sp., and *P. aeruginosa*, but notable antibacterial activity against *S. aureus*, *B. subtilis*, and *B. cereus*, with inhibition diameters ranging from 15 to 23 mm for leaf extracts and 17 to 18.5 mm for flower extracts. They also observed a complete absence of antifungal activity against *A. flavus* and *A. niger*. These studies underscore the importance of extraction methods, plant parts, and solvent polarity in determining antimicrobial potential. Unlike most previous investigations, which assessed only crude extracts, the present study demonstrates that fractionation allows clearer attribution of activity to chemical classes. Notably, the EtOAc and dichloromethane fractions exhibited the strongest antimicrobial effects, highlighting the importance of medium‐polarity metabolites rather than nonpolar lipids in determining bioactivity. The differences in antimicrobial activity among the fractions of *E. arborea* can be explained by their distinct chemical compositions, as revealed by GC–MS analysis (Table 3). The *n‐*hexane fraction, which showed no antibacterial activity and weak antifungal activity, was dominated by nonpolar compounds**,** including fatty acids (nonanoic, decanoic, dodecanoic, myristic, palmitic, and stearic acids), simple organic compounds (lactic acid, urea, glycerol), and 2,4‐di‐tert‐butylphenol. Although these compounds have been reported to exhibit mild antimicrobial properties through membrane destabilization [48, 49, 50], the absence of antibacterial activity in the present study could be explained by their relatively low concentrations as well as their limited intrinsic efficacy [51, 52]. Conversely, the presence of medium‐chain fatty acids such as nonanoic and decanoic acids may explain the slight antifungal activity observed against *C. albicans* and *F. oxysporum*, which are known to affect fungal membrane integrity [53]. In contrast, the dichloromethane fraction exhibited moderate antibacterial and antifungal activities, attributed to the presence of bioactive compounds such as benzoic acid and methyl caffeate, along with fatty acids. Benzoic acid is a known antimicrobial agent that disrupts cell membranes and inhibits microbial metabolism [54, 55], while methyl caffeate, a phenolic ester derived from caffeic acid, has been shown to possess antibacterial and antifungal activity, particularly against Gram‐positive bacteria [56, 57]. The presence of fatty acids such as palmitic acid and 1‐monopalmitin, as well as glycerolmonostearate, a surfactant, likely contributed synergistically to the improved activity of this fraction [50, 55]. The EtOAc fraction, which demonstrated the strongest antibacterial and antifungal effects, was enriched with phenolic acids and flavonoid‐type compounds**,** including protocatechuic acid, catechin, quinic acid, as well as fatty acids and glycerides. Phenolic acids are well documented for their antimicrobial effects, which include protein denaturation, cell wall disruption, metal ion chelation, and enzymatic inhibition [58, 59]. Catechin, in particular, is known for its strong antibacterial properties against Gram‐positive bacteria, likely due to its ability to damage bacterial membranes and inhibit nucleic acid synthesis [60]. Protocatechuic acid has also been reported to interfere with bacterial metabolism and cell wall synthesis [61]. The superior antimicrobial performance of the EtOAc fraction is therefore attributed to the synergistic effects of these bioactive compounds. The absence of activity against Gram‐negative bacteria is consistent with their intrinsic resistance mechanisms, particularly the lipopolysaccharide‐rich outer membrane that acts as a barrier to many antimicrobial agents, limiting their effectiveness [62, 63]. Even though bioactive phenolic compounds were present in the EtOAc fraction, their diffusion through the outer membrane of Gram‐negative bacteria is restricted. Regarding antifungal activity, *C. albicans* and *F. oxysporum* were more sensitive than filamentous fungi such as *A. niger* and *A. fumigatus*, likely due to differences in cell wall structure and composition. The simpler cell walls of *Candida* and *Fusarium* species make them more susceptible to compounds targeting cell membrane integrity or metabolic pathways [64, 65]. In contrast, the more complex hyphal cell walls of *A* *spergillus* species may confer greater resistance to antimicrobial agents [66, 67]. This study therefore provides novel insights into the compound‐specific basis of *E. arborea*'s antimicrobial properties and highlights the value of solvent fractionation for identifying the constituents responsible for bioactivity.

### 3.5 Molecular Docking

In this study, molecular docking analyses were performed to elucidate the potential mechanisms underlying the biological activities of the *E. arborea* EtOAc extract. This extract was selected for computational investigation because it exhibited the highest antioxidant and antibacterial activities in vitro, suggesting that it contains the most bioactive and pharmacologically relevant metabolites among all tested fractions.

The main objective was therefore to identify which metabolites within this extract contribute to its strong bioactivity by evaluating their binding affinity toward key enzymatic targets involved in oxidative stress and bacterial DNA replication. For each target, the binding energies and interaction profiles of all identified compounds were assessed, and only those displaying docking scores equal to or better than the reference inhibitors were retained for detailed interpretation.

The antibacterial assays performed in vitro revealed that the EtOAc extract of *E. arborea* exerted a notable inhibitory effect particularly against *S. aureus* and *B. subtilis*, which justified selecting these two bacterial strains for the computational investigation. DNA gyrase, composed of the GyrA and GyrB subunits, is an essential type II topoisomerase responsible for introducing negative supercoils into DNA during replication and transcription [68]. It is a validated antibacterial target because perturbing its activity leads to inhibition of DNA replication and, ultimately, bacterial cell death [12]. GyrA is primarily involved in DNA cleavage and strand passage, whereas GyrB supplies the ATP needed to drive the supercoiling process [69]. For this reason, both subunits were selected as docking targets to determine whether the metabolites from the EtOAc extract could interact with residues known to be critical for gyrase function and thereby explain the antibacterial properties measured experimentally.

**FIGURE 1:**
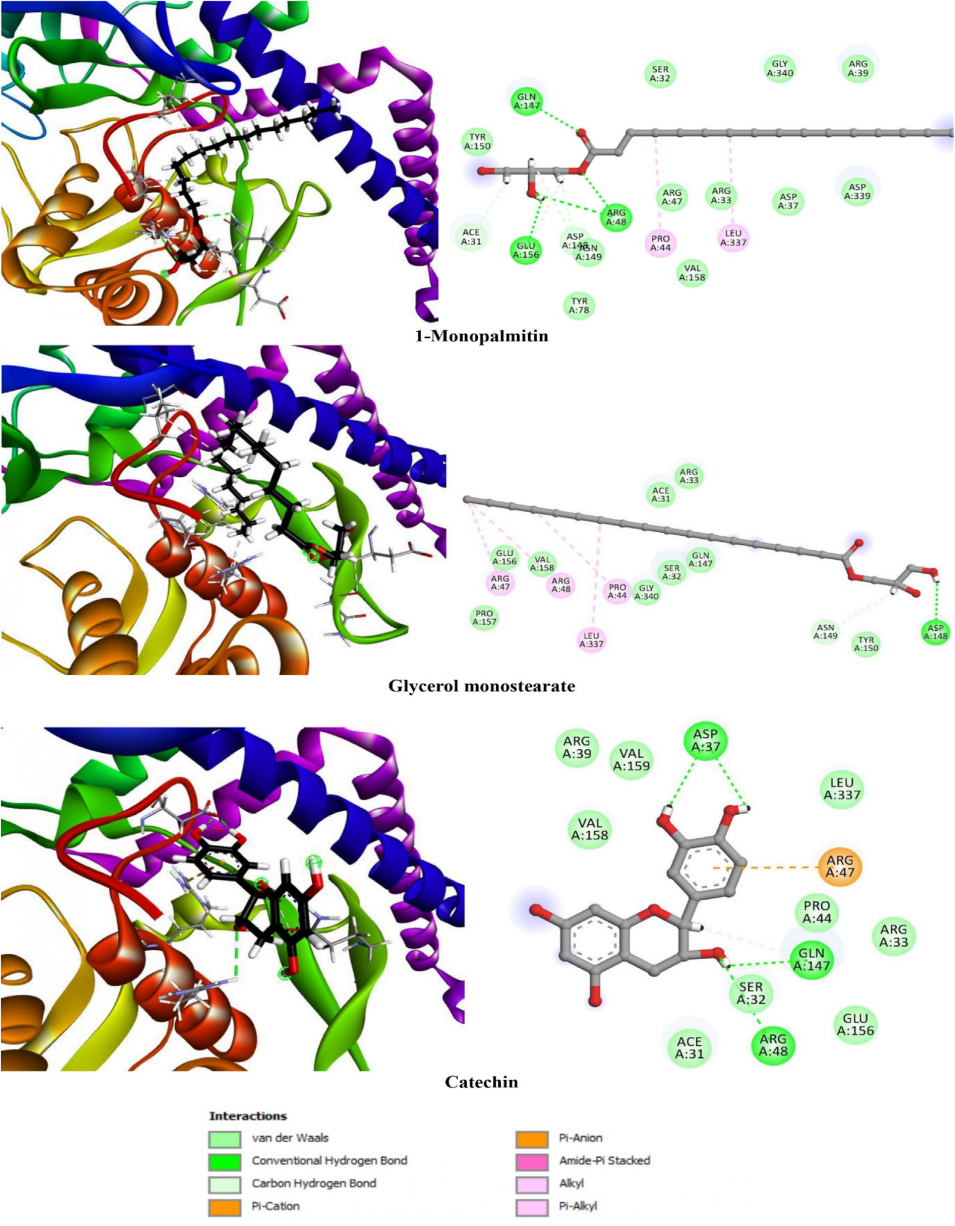
3D and 2D interaction diagrams of the best‐docked metabolites from *E. arborea* EtOAc extract within the active site of *B. subtilis* DNA GyrA (PDB: 4DDQ).

For *B. subtilis* GyrA (4DDQ), special attention was given to interactions within the quinolone‐binding pocket, as these residues are critical for DNA replication inhibition. The summarized results in Table 7 and Figure 1 highlight metabolites capable of establishing key stabilizing contacts similar to or stronger than ciprofloxacin. Only compounds presenting competitive binding energies were retained to streamline the discussion and emphasize the most relevant antibacterial candidates.

**TABLE 7:** Molecular docking results of *E. arborea* metabolites against bacterial DNA gyrase targets (GyrA: 4DDQ and GyrB: 3G75), compared with reference inhibitors.

The target protein	Ligand category	Ligand name	Binding energy, Kcal/mol	Hydrogen interactions, distance Å	Hydrophobic interactions	Electrostatic interactions
*B. subtilis* GyrA (4DDQ)	Best docked compounds	Ciprofloxacin (reference inhibitor)	−6.8	ARG33 (2.54), ASN149 (2.53), ASP148 (1.99), GLN147 (2.58), ASP148 (2.68, 2.81), GLU156 (2.86)	PRO44 (alkyl), PRO44 (π–alkyl)	GLU156 (attractive charge/electrostatic), ARG47 (salt bridge), ARG47 (salt bridge)
		1‐Monopalmitin	−7.1	ARG48 (2.72, 2.28), GLN147 (2.34), GLU156 (1.96), ASP148 (2.43), GLU156 (2.79), ASP148 (2.88), ACE31 (2.41)	PRO44 (alkyl), LEU337 (alkyl)	—
		Glycerol monostearate	−6.9	ASP148 (1.89), ASN149 (2.75)	PRO44 (alkyl), ARG47 (alkyl), ARG48 (alkyl), LEU337 (alkyl)	—
		Catechine	−6.8	ARG48 (2.97), ASP37 (1.89), ASP37 (1.81), GLN147 (2.05), GLN147 (2.68)	—	ARG47 (π–cation)
*S. aureus* GyrB (3G75)	Co‐crystallized ligand	B48	−6.6	ASP81 (2.69), THR173 (4.15)	VAL79 (alkyl), ILE175 (alkyl), ILE86 (alkyl), ILE86 (π–alkyl), PRO87 (π–alkyl)	ARG84 (π–cation), GLY85/ILE86 (amide–π stacked)
	Best docked compounds	Glycerol monostearate	−7.6	ASN54 (2.11, 2.74), ASN54 (2.34, 2.62), SER129 (2.49), SER129 (3.09), GLU58 (2.48)	ARG84 (alkyl), PRO87 (alkyl), LEU86 (alkyl)	—
		1‐Monopalmitin	−7.0	ASN54 (2.53), ASP81 (2.32)	ARG84 (alkyl), PRO87 (alkyl), LEU86 (alkyl), TYR63 (π–alkyl)	—
		Catechin	−6.9	ASP81 (2.01), ASP81 (2.01), GLU58 (2.67)	ASN54 (amide–π stacked), PRO87 (alkyl), ARG84 (π–alkyl), PRO87 (π–alkyl), ILE86 (π–alkyl)	ARG84 (π–cation), GLU58 (π–anion)

The reference ligand ciprofloxacin displayed a binding affinity of −6.8 kcal/mol, stabilized through a dense hydrogen bonding network with ARG33, ASN149, ASP148, GLN147, and GLU156, as well as salt‐bridge interactions with ARG47. These interactions are characteristic of well‐established GyrA inhibitors, validating the docking protocol. Several extract‐derived molecules showed equal or stronger affinities than ciprofloxacin. 1‐Monopalmitin exhibited the best binding score (−7.1 kcal/mol), forming seven hydrogen bonds with critical residues such as ARG48, GLN147, ASP148, and GLU156. These interactions anchor the ligand deeply within the catalytic pocket. Additionally, hydrophobic contacts with PRO44 and LEU337 stabilize its long aliphatic chain, supporting a dual polar–hydrophobic binding mode. The absence of electrostatic interactions suggests that binding is primarily driven by hydrogen bonding and van der Waals complementarity. Glycerol monostearate (−6.9 kcal/mol) also showed strong affinity, forming hydrogen bonds with ASP148 and ASN149, both residues involved in stabilizing quinolone inhibitors. Its interaction pattern is dominated by hydrophobic anchoring via ARG47, ARG48, PRO44, and LEU337, consistent with the behavior of large amphiphilic ligands within the GyrA pocket. Catechin displayed a similar affinity to ciprofloxacin (−6.8 kcal/mol), but with a distinct interaction mode. It formed five hydrogen bonds with ASP37, GLN147, and ARG48, while establishing a key π–cation interaction with ARG47, a crucial residue for quinolone stabilization. This suggests that catechin may mimic some features of aromatic GyrA inhibitors, despite its polyphenolic nature. The GyrA docking study indicates that fatty acid derivatives (monopalmitin, glycerol monostearate) and polyphenols (catechin) from *E. arborea* have the capacity to bind effectively to the catalytic pocket of GyrA, with affinities comparable to the reference drug ciprofloxacin. The diversity of their binding modes, hydrogen bonding, hydrophobic packing, and π interactions highlights the potential multi‐target antibacterial behavior of the extract.

**FIGURE 2:**
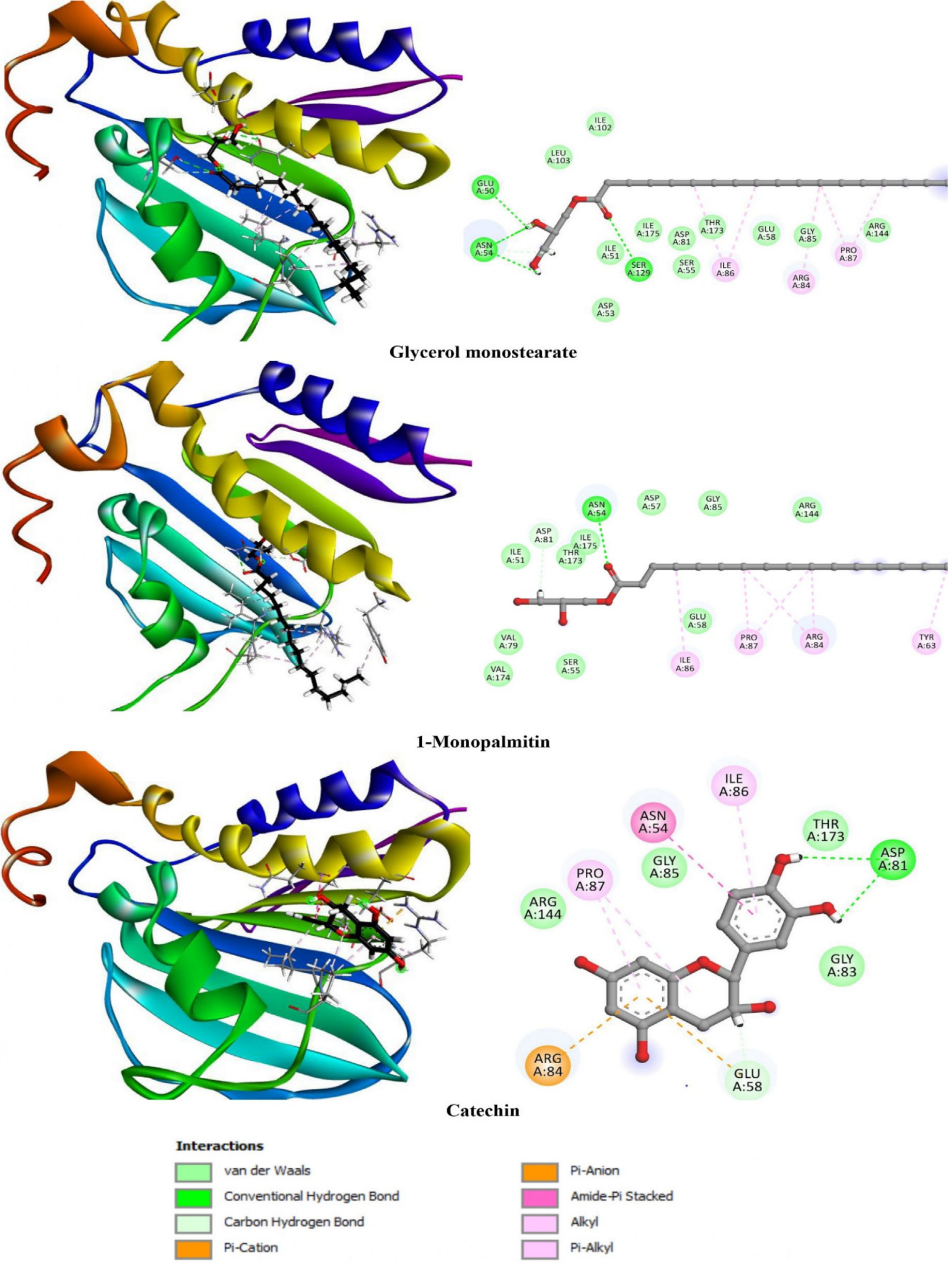
3D and 2D interaction profiles of the top docked metabolites from *E. arborea* EtOAc extract within the active site of *S. aureus* DNA GyrB (PDB: 3G75).

In the case of *S. aureus* GyrB (3G75), docking results revealed differential binding behaviors among the tested metabolites. Their interaction profiles and energy scores are presented in Table 7 and Figure 2, and only those surpassing or matching the affinity of the co‐crystallized ligand B48 were selected for detailed discussion. This selection allows for clearer identification of compounds with potential to disrupt ATP‐dependent DNA supercoiling, thereby contributing to the antibacterial activity observed experimentally. The co‐crystallized ligand B48 achieved a binding score of −6.6 kcal/mol. As expected for ATP‐binding site inhibitors, B48 interacts primarily through hydrogen bonds with ASP81 and THR173, supported by hydrophobic contacts involving VAL79, ILE175, ILE86, and PRO87. π–cation interactions with ARG84 and amide–π stacking with GLY85/ILE86 contribute further to binding stability. Among the extract components, glycerol monostearate showed the strongest affinity (−7.6 kcal/mol), outperforming the reference ligand. This molecule formed multiple hydrogen bonds with ASN54, SER129, and GLU58, residues located within the nucleotide binding pocket. It also engaged in hydrophobic interactions with ARG84, PRO87, and LEU86, suggesting a strong anchoring mechanism that occupies the ATP‐binding groove. The absence of electrostatic interactions indicates that stabilization arises mainly from polar and hydrophobic complementarity. 1‐Monopalmitin also bound favorably (−7.0 kcal/mol), showing key hydrogen bonds with ASN54 and ASP81, similar to those established by B48. Its binding is further strengthened by several alkyl and π‐alkyl interactions with ARG84, PRO87, and LEU86, suggesting that long‐chain glycerides may interfere with ATP‐dependent GyrB function via hydrophobic occlusion of the binding cavity. Catechin displayed a binding score of −6.9 kcal/mol and exhibited a more complex interaction profile. It formed several hydrogen bonds with ASP81 and GLU58, while engaging in π‐cation, π‐anion, and amide–π interactions with ARG84, GLU58, and ASN54, respectively. These interactions mimic the binding behavior of known ATP‐competitive inhibitors of GyrB, highlighting catechin as a promising scaffold for antibacterial activity.

**FIGURE 3:**
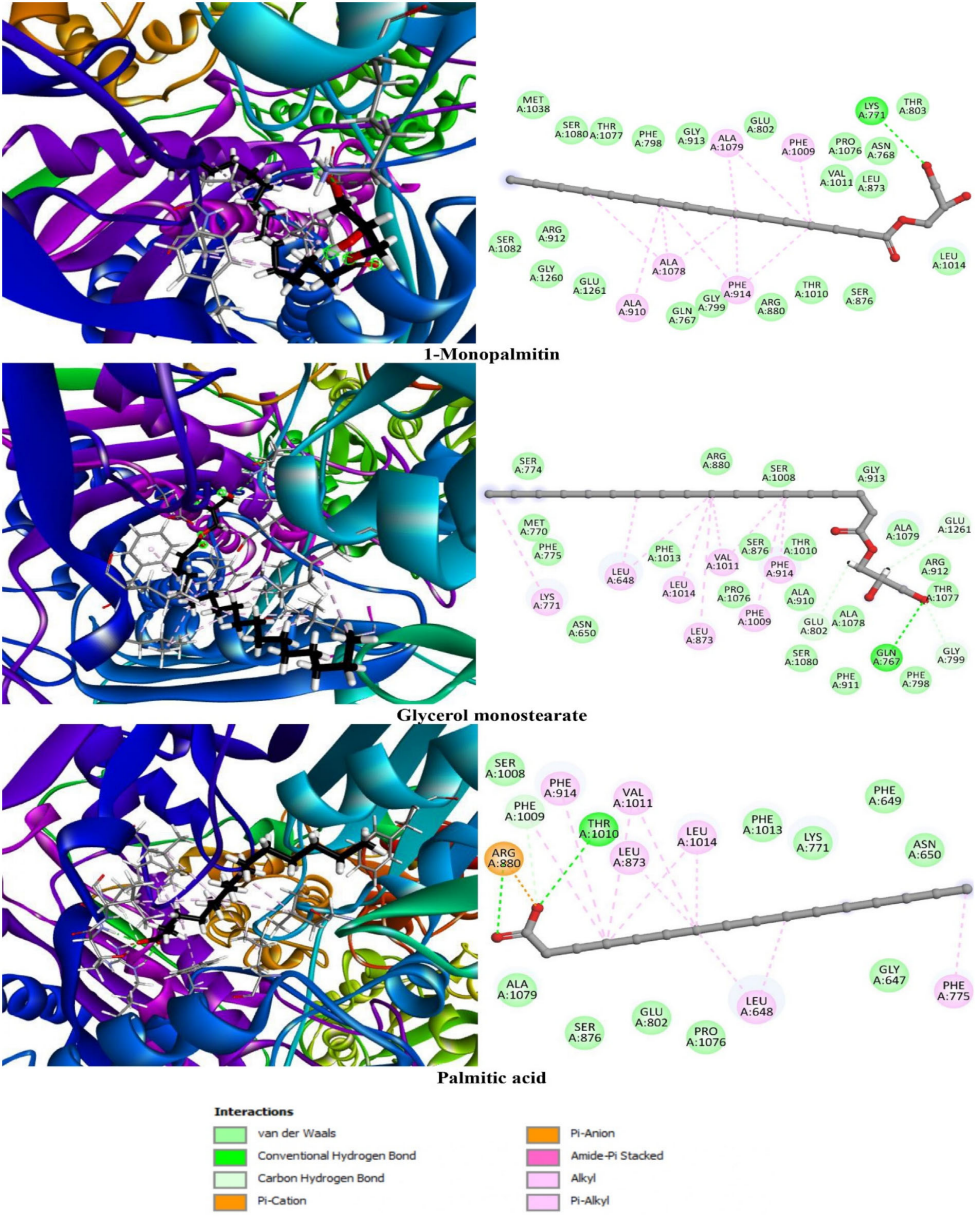
3D and 2D binding interaction diagrams of selected metabolites from *E. arborea* EtOAc extract docked into the active site of Xanthine oxidase (PDB: 1N5X).

The antioxidant potential of the EtOAc extract was further explored through molecular docking against xanthine oxidase (XO), an enzyme well known for its central role in the production of reactive oxygen species via the oxidation of hypoxanthine to uric acid [65]. Inhibiting XO is therefore considered a key strategy for reducing oxidative stress, which aligns directly with the strong antioxidant activity demonstrated by the extract in vitro. For this reason, XO was selected as a relevant molecular target to identify which phytoconstituents may contribute to the observed biological effect. The docking outcomes are summarized in Table 8 and illustrated in Figure 3, providing a comparative overview of the interaction profiles of the most promising compounds relative to the co‐crystallized inhibitor TEI‐6720.

**TABLE 8:** Docking results of selected *E. arborea* metabolites against xanthine oxidase (XO, PDB ID: 1N5X), compared with the co‐crystallized inhibitor TEI‐6720.

The target protein	Ligand category	Ligand name	Binding energy, kcal/mol	Hydrogen interactions	Hydrophobic interactions	Electrostatic/metal interactions
XO (1N5X)	Co‐crystallized ligand	(TEI‐6720)	−7.5	ASN768 (1.97), LYS771 (2.50, 2.63), ARG880 (3.77), THR1010 (1.76, 2.16), PHE1009 (2.64), PRO1076 (2.37),	PHE1009 (π–π stacked), PHE914 (π–π T‐shaped), LEU873 (alkyl), VAL1011 (alkyl), LEU648 (alkyl), ALA1079 (π–alkyl)	GLU802 (salt bridge), ARG880 (attractive charge), PHE914 (pi‐cation), PHE1009 (pi‐anion)
	Best docked compounds	1‐Monopalmitin	−8.2	LYS771 (3.09, 2.90)	ALA910 (alkyl), ALA1078 (alkyl), ALA1079 (alkyl), PHE914 (π–alkyl), PHE1009 (π–alkyl)	—
		Glycerol monostearate	−8.0	GLN767 (2.16), GLY799 (2.65), GLU802 (2.98), GLU1261 (2.50)	VAL1011 (alkyl), LYS771 (alkyl), LEU648 (alkyl), LEU873 (alkyl), LEU1014 (alkyl), PHE914 (π–alkyl), PHE1009 (π–alkyl)	
		Palmitic acid	−7.5	ARG880 (2.18), THR1010 (2.04, 1.86), PHE1009 (2.79)	VAL1011 (alkyl), VAL1011 (alkyl), LEU648 (alkyl), LEU648 (alkyl), LEU873 (alkyl), LEU1014 (alkyl), PHE775 (π–alkyl), PHE914 (π–alkyl), PHE1009 (π–alkyl)	ARG880 (attractive charge)

*Note:* The docking analysis performed on xanthine oxidase (XO, PDB ID: 1N5X) revealed that several constituents of the *E. arborea* EtOAc extract exhibited binding affinities comparable to, or stronger than, the co‐crystallized inhibitor TEI‐6720 (−7.5 kcal/mol). These findings strongly support the hypothesis that the antioxidant activity of the extract may be associated with an ability to modulate XO, a key enzyme responsible for superoxide anion generation during purine metabolism.

The co‐crystallized ligand TEI‐6720 displayed a rich hydrogen‐bonding network involving ASN768, LYS771, ARG880, THR1010, PHE1009, and PRO1076, in addition to multiple hydrophobic contacts with PHE1009, PHE914, LEU873, VAL1011, LEU648, and ALA1079. Importantly, TEI‐6720 also established strong electrostatic interactions, including a salt bridge with GLU802, attractive charge interactions with ARG880, and π–cation/π–anion interactions involving PHE914 and PHE1009. This interaction profile is characteristic of potent XO inhibitors, confirming the validity of the docking protocol. Among the docked metabolites, 1‐monopalmitin showed the highest affinity (−8.2 kcal/mol), outperforming the reference ligand. Its stabilization within the active site was mainly hydrophobic, through extensive alkyl and π–alkyl contacts (ALA910, ALA1078, ALA1079, PHE914, PHE1009). In addition, the formation of two hydrogen bonds with LYS771 indicates a favorable anchoring mechanism near the molybdenum‐pterin cavity. Although monoacylglycerols are not classical XO inhibitors, their bulky hydrophobic chains appear to engage deeply with the lipophilic regions of the binding site, suggesting a steric blocking mechanism that may prevent substrate access to the catalytic core. Glycerol monostearate also exhibited strong affinity (−8.0 kcal/mol), supported by four hydrogen bonds involving GLN767, GLY799, GLU802, and GLU1261, residues located near the FAD‐binding and molybdenum centers. Additionally, the compound displayed numerous stabilizing hydrophobic interactions with VAL1011, LYS771, LEU648, LEU873, LEU1014, PHE914, and PHE1009. Unlike monoacylglycerols that rely mainly on hydrophobic packing, glycerol monostearate combines both polar anchoring and hydrophobic embedding, which may enhance its ability to interfere with XO activity. The involvement of GLU802, a residue participating in the catalytic environment, suggests that this compound may influence the redox processes required for xanthine hydroxylation. Palmitic acid displayed a binding affinity equal to that of the co‐crystallized inhibitor (−7.5 kcal/mol). Despite its simple structure, it formed several hydrogen bonds with ARG880 and THR1010, as well as a carbon H‐bond with PHE1009, indicating that fatty acids can engage in selective polar interactions within the active pocket. The compound also showed extensive hydrophobic contacts with VAL1011, LEU648, LEU873, LEU1014, and aromatic residues (PHE775, PHE914, PHE1009), which help stabilize the aliphatic chain. Notably, palmitic acid exhibited an attractive charge interaction with ARG880, an important residue located near the substrate channel, suggesting the potential to alter substrate positioning or electron transfer pathways. Overall, the docking results indicate that lipid‐based constituents of *E. arborea*, particularly 1‐monopalmitin and glycerol monostearate, interact strongly with key catalytic and structural residues of XO through a combination of hydrogen bonding, hydrophobic packing, and electrostatic complementarity. Their higher or equal binding affinities compared to TEI‐6720 suggest that these molecules may contribute significantly to the antioxidant potential of the EtOAc extract by reducing superoxide anion production through XO inhibition. The convergence of these results demonstrates that both complex amphiphilic molecules (monoacylglycerols) and simple fatty acids (palmitic acid) have the capacity to bind efficiently to the XO active site. This may explain the strong antioxidant activity associated with the extract and highlights the relevance of lipidic phytoconstituents in modulating redox‐related enzymes. However, further in vitro enzymatic assays and kinetic analyses are necessary to validate the docking predictions and determine the precise inhibitory mechanisms.

## 4 Conclusion

In conclusion, this study provides a thorough investigation of *E. arborea*, demonstrating its considerable potential as a source of bioactive compounds. The EtOAc fraction, in particular, was identified as chemically rich, containing high levels of polyphenols, flavonoids, and flavonols, which were further confirmed by GC–MS analysis. This chemical richness translated into pronounced biological activities, with the EtOAc extract exhibiting strong antioxidant effects across multiple assays and notable antimicrobial activity against both bacterial and fungal strains. The molecular docking analysis provided valuable insight into the mechanisms that may underlie the antioxidant and antibacterial activities of the *E. arborea* EtOAc extract. By targeting xanthine oxidase as well as the bacterial DNA gyrase subunits GyrA and GyrB, the simulations revealed that several phytoconstituents interact strongly with key catalytic residues, with binding affinities comparable to or exceeding those of the reference inhibitors. Altogether, the docking results offer a coherent molecular explanation that complements the experimental assays, confirming that specific metabolites within the extract have the potential to modulate oxidative processes and interfere with bacterial DNA replication pathways. These insights reinforce the therapeutic relevance of *E. arborea* and highlight promising candidates for future pharmacological investigation.

To fully uncover the therapeutic potential of *E. arborea*, future investigations should employ complementary analytical techniques and bioassay‐guided isolation to further characterize potential bioactive constituents and validate their mechanisms of action.

## Author Contributions

Conceptualization: Amina Bramki, Ouided Benslama, and Marco Masi. Methodology: Amina Bramki, Ouided Benslama, Zineb Mokhbi, Selsabile Nedjaoum, and Marco Masi. Software: Ouided Benslama, Fatima Zohra Makhlouf, and Marco Masi. Validation: Amina Bramki, Ouided Benslama, and Marco Masi. Formal analysis: Maria Michela Salvatore and Marco Masi. Investigation: Amina Bramki, Ouided Benslama, Maria Michela Salvatore, and Marco Masi. Resources: Amina Bramki, Chawki Bensouici, and Marco Masi. Data curation: Ouided Benslama and Marco Masi. Writing – original draft preparation: Amina Bramki, Ouided Benslama, Fatima Zohra Makhlouf, and Marco Masi. Writing – review and editing: Amina Bramki, Ouided Benslama, Anna Andolfi, Maria Michela Salvatore, and Marco Masi. Visualization: Amina Bramki, Ouided Benslama, and Marco Masi. Supervision: Amina Bramki and Marco Masi. Project administration: Amina Bramki and Marco Masi. Funding acquisition: Chawki. Bensouici and Marco Masi.

## Conflicts of Interest

The authors declare no conflicts of interest.

## Data Availability

The data that support the findings of this study are available from the corresponding author upon reasonable request.
